# A pragmatic approach to monitor and evaluate implementation and impact of differentiated ART delivery for global and national stakeholders

**DOI:** 10.1002/jia2.25080

**Published:** 2018-03-14

**Authors:** Peter D Ehrenkranz, Jesus MG Calleja, Wafaa El‐Sadr, Ade O Fakoya, Nathan Ford, Anna Grimsrud, Kate L Harris, Suzanne L Jed, Daniel Low‐Beer, Sadhna V Patel, Miriam Rabkin, William John Reidy, Annette Reinisch, George K Siberry, Leigh A Tally, Isaac Zulu, Irum Zaidi

**Affiliations:** ^1^ Global Development Bill & Melinda Gates Foundation Seattle WA USA; ^2^ Department of HIV and Hepatitis WHO Geneva Switzerland; ^3^ ICAP at Columbia University Columbia University Mailman School of Public Health New York NY USA; ^4^ Technical Advice and Partnerships Department The Global Fund to Fight AIDS, Tuberculosis and Malaria Geneva Switzerland; ^5^ HIV Programmes & Advocacy International AIDS Society Cape Town South Africa; ^6^ Office of the U.S. Global AIDS Coordinator and Health Diplomacy U.S. Department of State Washington DC USA; ^7^ Global Division, HIV/AIDS Bureau U.S. Health Resources and Services Administration Rockville MD USA; ^8^ Division of Global HIV and TB Center for Global Health Centers for Disease Control and Prevention Atlanta GA USA

**Keywords:** HIV, differentiated care, differentiated service delivery, monitoring and evaluation, efficiency, productivity, health care worker experience, patient experience

## Abstract

**Introduction:**

The World Health Organization's (WHO) recommendation of “Treat All” has accelerated the call for differentiated antiretroviral therapy (ART) delivery, a method of care that efficiently uses limited resources to increase access to HIV treatment. WHO has further recommended that stable individuals on ART receive refills every 3 to 6 months and attend clinical visits every 3 to 6 months. However, there is not yet consensus on how to ensure that the quality of services is maintained as countries strive to meet these standards. This commentary responds to this gap by defining a pragmatic approach to the monitoring and evaluation (M&E) of the scale up of differentiated ART delivery for global and national stakeholders.

**Discussion:**

Programme managers need to demonstrate that the scale up of differentiated ART delivery is achieving the desired effectiveness and efficiency outcomes to justify continued support by national and global stakeholders. To achieve this goal, the two existing global WHO HIV treatment indicators of ART retention and viral suppression should be augmented with two broad aggregate measures. The addition of indicators measuring the frequency of (1) clinical and (2) refill visits by PLHIV per year will allow evaluation of the pace of scale up while monitoring its overall effect on the quality and efficiency of services. The combination of these four routinely collected aggregate indicators will also facilitate the comparison of outcomes among facilities, regions or countries implementing different models of ART delivery. Enhanced monitoring or additional assessments will be required to answer other critical questions on the process of implementation, acceptability, effectiveness and efficiency.

**Conclusions:**

These proposed outcomes are useful markers for the effectiveness and efficiency of the health system's attempts to deliver quality treatment to those who need it—and still reserve as much of the available resource pool as possible for other key elements of the HIV response.

## 1 Introduction: The potential of differentiated service delivery and the need to measure its impact

The World Health Organization's (WHO) recommendation that all people living with HIV (PLHIV) should start antiretroviral therapy (ART) as soon as possible—“Treat All” 1—accelerated the need to more effectively use the limited available resources to increase access to HIV treatment while ensuring quality. To implement this ambitious agenda, countries must both scale up access to differentiated service delivery models while simultaneously building capacity to collect and use data to improve programming. Without these endeavours, the target of sustainably doubling the numbers of people on effective treatment worldwide from 19 million to more than 36 million, and ensuring sustained viral suppression for all, will not be achieved by the year 2030 2.

Differentiated service delivery (DSD) is a public health approach that responds to the increasing diversity of needs of PLHIV 3, 4. DSD is not new. Rather, it builds on the foundation of well‐known interventions—decentralization, task sharing, integration, extended refills, peer support—in an intentional strategy that targets the traditional public health response at a demographic group or geography in a manner that is responsive to the needs of the clients receiving care. While DSD concepts can be applied across the spectrum of HIV services—from prevention through viral suppression—*differentiated ART delivery* addresses the treatment needs of PLHIV on lifelong ART 4.

Differentiated ART delivery has received broad support from stakeholders. WHO strongly recommends that a stable individual on ART receive less frequent ART refills (3 to 6 monthly rather than current standard of monthly refills) and less frequent clinic visits (3 to 6 monthly) 1. National HIV programmes have adapted various differentiated ART delivery approaches to their contexts 5, 6, 7. Clients and their families appreciate that these approaches are responsive to their evolving requests for more client‐centred, and potentially less burdensome, management. Healthcare workers (HCWs) see an opportunity to provide improved care for the clients and communities they serve while decreasing the numbers of patients in their daily queue. International agencies, particularly the Global Fund to Fight AIDS, Tuberculosis and Malaria and United States President's Emergency Plan for AIDS Relief (PEPFAR), the two largest donors to the HIV response, are motivated at a time of constrained resources and ambitious goals to demonstrate that their resources are being used to do more, better, with less 8, 9, 10, 11, 12. The measurement of effective and efficient allocation and use of resources to achieve quality care therefore represent a shared interest across the board—from recipients of care to health workers, programme managers and funders. However, the need to demonstrate the impact of differentiated models and improve their implementation must be balanced with the need to minimize additional burdens on frontline workers.

By defining the expectations of donor and normative agencies, this commentary seeks to describe a pragmatic approach for monitoring and evaluation (M&E) of differentiated ART delivery. It encourages countries to utilize WHO strategic information guidelines and M&E indicators for ART programmes 13, 14. It then suggests that countries introduce two new indicators to monitor the pace of differentiated ART delivery scale up and enhance comparisons across countries. Specifically, it suggests the use of broad (not DSD model‐specific) aggregate outcome indicators that can be collected routinely and will remain applicable even as model implementation evolves. It then identifies the value of augmenting routine data collection with targeted investigations that can provide more robust assessments of implementation scale‐up, effectiveness, and efficiency. The intention is to define the minimum measures that should be collected to compare the pace and quality of outcomes of scale up of differentiated ART delivery at subnational, national, or global levels. A complementary discussion on patient‐ and programme‐level monitoring of differentiated service delivery for HIV has been published elsewhere 15.

## 2 Discussion

### 2.1 General guidance for M&E of differentiated ART delivery: at facilities, districts, national and global levels

General guidance on the M&E of differentiated ART delivery can be found in the recent WHO guidelines, *Person‐Centred HIV Patient Monitoring and Case Surveillance* 14. This guidance recommends a shift from collecting aggregated service‐level data (e.g. the number of HIV tests provided) to individual level data focused on a client's receipt of various linked services and an emphasis on ensuring that the data are used to improve patient outcomes. It describes the patient data to be collected at service delivery points in all facility and community settings—including eligibility for, enrolment in, and retention in various treatment models, as well as clinic, pharmacy and laboratory service information. This information will need to be recorded in appropriate charts and registers, preferably using unique identifiers and electronic data systems to enable longitudinal client management across multiple settings. While the data elements from client charts as well as community or facility‐based tools may be needed for patient care or quality improvement (QI) activities at the service level, only the most essential (i.e. treatment regimen, stopped treatment, lost to follow up, virologic suppression, death, etc.) need to be aggregated for programmatic reviews every 6 to 12 months.

At the district, national, or global levels, such aggregated routine indicators already captured in health management information systems should be used to measure the outcomes of differentiated ART delivery implementation. WHO's 2015 Consolidated Strategic Information Guidelines 13 give clear guidance on M&E for these outcomes, specifying fifty national and ten global indicators (selected from among the fifty) that all countries should collect. Any additional changes to this list should be made cautiously and should maintain an approach that does not require model‐specific indicators.

Minimizing, simplifying and standardizing any new M&E requirements is critical when introducing an innovation like differentiated ART delivery. The innovation is most likely to be adopted and sustained within clinical practice if it proves to be intrinsically rewarding for the HCWs by making their jobs easier or more gratifying 16. For example, if data are available for a facility to use in QI programming that leads to more uptake of differentiated ART models, HCWs may see that the combination of differentiated ART and pragmatic data systems leads to both better health outcomes for their clients and lighter workloads for themselves. However, if the M&E requirements are onerous or not tied to such a positive feedback loop, there is risk that HCWs may be deterred from enrolling patients in different models of ART delivery.

### 2.2 The addition of two aggregate indicators enables measurement of scale up of differentiated ART delivery at national and global levels

Measuring progress towards scale up of differentiated ART delivery requires that the existing global WHO HIV treatment indicators of ART retention and viral suppression 13 be supplemented with only two additional aggregate indicators, which are the result of a consultative process among international and national experts:

- Number of clinical visits performed/individual PLHIV currently on treatment/12 month period
- Number of visits at which medication pickup occurs/individual PLHIV currently on treatment/12 month period

While some imprecision in the data used for calculating numerators and denominators is inevitable, use of standardized definitions currently under development by normative agencies will help ensure consistency in terms of how the data are derived.

A key advantage of these two indicators is that they recognize the potential variability within a differentiated ART delivery model when it is implemented in different settings. For example, a client‐managed group model in which members distribute ART refills to each other (often referred to as a “community adherence group” or CAG) may have the Mozambique‐originated design of six people who rotate visits to the facility 17, 18. In this model, a PLHIV would have a clinical visit every 6 months and a refill every 1 month. Other CAGs may have ten members attend their 6 monthly or annual clinical visit together or have just two members, a married couple, who alternate visits 5. The same variability can be expected of other models of differentiated ART delivery, including HCW‐managed groups like adherence clubs; facility‐based individual models often known as fast track refills; or out‐of‐facility individual models that utilize community distribution points or home delivery 19.

A second advantage of the generic indicators is that they use existing records to provide a high‐level indication of the frequency (and therefore the intensity) of the PLHIV's interaction with the health system. They do not, however, require aggregation of the detailed record of each PLHIV's clinical status or the model of care in which s/he is enrolled at all times (which will change as a client cycles in and out of models and between facilities as his/her health needs change). The clinical visit indicator would require access to service level registries or, if available, electronic records. With appropriate interoperability between information systems, the refill indicator should be based on existing pharmacy records—as is the existing WHO indicator “ART adherence proxy.”

Lastly, by monitoring progress towards a decrease in the intensity of interaction between PLHIV and the healthcare system, the generic indicators will provide early insight into the consequences of countries attempting to use health system, funder and client resources more efficiently. Rather than requiring regular (and expensive) additional costing studies, the trend of use of health system and client resources can feasibly be followed by a proxy: “average number of clinic visits/PLHIV currently on treatment in the last year.” Changes in resource use must then be compared with existing national indicators like retention and viral suppression to ensure that quality is maintained even as differentiated models are scaled up. While aggregated data masks heterogeneity, such comparisons will facilitate important early warnings if a country's focus on efficiency is leading to a deterioration of client outcomes.

### 2.3 Countries with robust medical records systems have more options for evaluation, stratification and target setting

The proposed indicators provide a minimum starting point for evaluations of the fidelity of implementation and impact of differentiated ART delivery. Countries should be encouraged to use and strengthen the data systems that they have available (electronic, paper, or a combination) to obtain a baseline of implementation and then monitor the impact of scale up. For example, if pharmacy data are available, a programme could assess how many patients are receiving quantities of medication sufficient to fill the period between refills. National programmes with more robust medical records systems can further stratify indicators of coverage or outcomes by eligibility for enrolment in a model, facility, key clinical information (eg, CD4 count at entry to care), gender, age, specific population, and location (rural/urban). In addition, a country could set evidence‐based targets for an appropriate level of differentiation based on its context and national guidelines:

- One country's guidelines could be that PLHIV 4, including children greater than 2 years old, adolescents, adults and specific populations who are well, in care for six or more months, and have suppressed viral load are eligible for less intense models of care. This country could set its target at: *80% of all PLHIV with most recent viral load less than 1000* *copies/mL should receive ART refills no less than every 3 months and attend an HIV clinical visit no more than once every 6 months*.
- Another country, with low coverage of viral load, supply chain concerns, or other systems challenges, could choose to adopt simpler national guidelines that prescribe a combined clinical/refill visit every 3 months for every adult patient. It could therefore set a target that *70% of adult PLHIV, regardless of viral load or clinical status, should have a combined clinical/refill visit every 3 months*.

In both cases, these targets account for the reality that some clients—those newly initiating ART, presenting or returning to care with advanced disease, or due to personal preference—may require different approaches that would fall outside of the mainstream target. Even so, there are potential unintended consequences to such targets. To mitigate them, countries need to carefully consider the context in which their ART delivery programmes are operating and pay close attention to the core quality indicators of retention and viral suppression. In settings where monitoring systems are not robust enough to provide a desired granularity, investigations into detailed questions can be achieved through other means.

### 2.4 Enhanced monitoring or additional investigations are required for more specific questions on process, patient and healthcare worker experience, scale of implementation, effectiveness, cost and efficiency

A critical goal of any Ministry of Health seeking to improve its ART delivery should be to strengthen the collection, analysis, and use of data through its routine data systems. If additional data about the implementation, outcomes, and impact of differentiated ART delivery beyond routine data collection are deemed critical, then other options for evaluation include:

- enhanced monitoring at all or a sample of sites—this process involves active collection of routine data on a more frequent schedule and/or collection of additional data both to trigger timely identification of implementation or outcome challenges that need corrective action and to monitor change in such processes and outcomes 20
- incorporation of key questions into other large‐scale surveys (e.g. Demographic Health Surveys, or Population‐based HIV Impact Assessments) that align with monitoring indicators
- cohort monitoring
- case‐based reporting 21, or
- other targeted studies

Any of these investigations could be designed to answer detailed implementation science questions that go beyond the scope of routine reporting indicators, including experience of PLHIV and HCWs with the new models, detailed clinical outcomes, and efficiency and costs of healthcare delivery (Table 1) 22. While evaluations of clinical outcomes, costs and efficiency are not uncommon, assessing the personal experience of patients and HCWs has rarely been done. It will require conduct of mixed methods studies that use exit interviews or brief satisfaction questionnaires (provided in person or electronically) to capture HCW and PLHIV impressions of clinical care offered/provided, respect, stigma and discrimination, waiting time, infrastructure, availability of commodities, transport costs and overall satisfaction. Use of similarly defined indicators and/or protocols across multiple settings would facilitate comparisons of these qualitative outcomes, especially if results were disaggregated by age and sex. One could hypothesize that regions with a high uptake of differentiated models will see patients and healthcare workers more satisfied with their experience with the healthcare system, better retention and viral load suppression, reduced morbidity and mortality as clinic services are directed to managing patients with advanced HIV disease, and lower patient and provider costs than those with less uptake of the models.

**Table 1:** Four domains and proposed indicators to assess differentiated ART delivery using routine data supplemented with special studies

Domain	Indicators	Sources of information
Coverage of differentiated ART delivery *(newly proposed indicators)*	**# of visits at which medication pickup occurs/PLHIV currently on treatment/12 month period** a **# of clinical visits/PLHIV currently on treatment/12 month period** a	Routine program data
Experience of PLHIV and HCWs	PLHIV experience, including experience of those who disengaged from treatment HCW experience	Facility and community surveys
Clinical outcomes	**# and % PLHIV virally suppressed/12 month period** a **# and % PLHIV retained in care/12 month period** a # and % PLHIV lost to follow up/12 month period # and % PLHIV who died/12 month period	Routine program data
Cost and efficiency of health care delivery from the perspective of the patient and the provider	Mean time for a clinical consultation/PLHIV/visit Mean total time spent by the patient to receive HIV treatment services (including transportation and waiting)/PLHIV/6 months period Mean out‐of‐pocket cost to patient to receive HIV treatment services (including clinic, medication, transportation)/PLHIV/6 months period # of PLHIV receiving clinical consultations/day/HCW # of patients (of any condition other than HIV) receiving clinical consultations/day/HCW^b^ Mean cost of treatment services from a provider perspective/PLHIV/year Mean cost of treatment services from a provider perspective/virally suppressed PLHIV/year	Routine program data Facility and community surveysAnalyses of financial records

a The minimum indicators that should be routinely collected from amongst an entire ART cohort to monitor the pace and quality of scale up of differentiated ART delivery at subnational, national, or global levels are in bold. Each should be disaggregated by age and sex. Other proposed indicators may be collected routinely in some contexts, but will most likely require special studies to ensure accuracy.

b An additional productivity indicator that will allow determination of whether or not scale up of DSD frees up HCWs to see patients with conditions other than HIV.

## 3 Conclusions

The ultimate metrics of a country's success in HIV epidemic control are declines in incidence of new infections and mortality. The contributions of treatment programmes, and differentiated ART delivery itself, to these goals are best measured via: the WHO indicators of retention and viral suppression, HIV‐associated mortality indicators, and healthcare utilization/cost indicators such as mean number of clinic visits/PLHIV/year. These outcomes are markers for the efficiency and effectiveness of the health system's attempts to deliver quality treatment to those who need it—and still reserve as much of the available resource pool as possible for other key elements of the HIV response.

Scaling up differentiated ART delivery models is an important lever for achieving these objectives. To understand the models’ potential effects, though, efforts to measure outcomes and improve impact and efficiency should be person‐centred at the facility level and require minimal changes to be aggregated at the national and global levels. Supplementary efforts to collect key process, outcome, cost and efficiency indicators to monitor and improve implementation should occur in parallel only when truly needed.

## Competing interests

None of the authors have competing interests to declare.

## Authors’ contributions

The concept for this commentary was developed by JC, PE, WE, AF, NF, AG, KH, SJ, DL, SP, AR, MR, WR, GS, TL, IZaidi, IZulu. PE wrote the first draft. All authors contributed and approved the final version.

## Funding

Technical expertise for this document was supported in part by the U.S. President's Emergency Plan for AIDS Relief (PEPFAR), the U.S. Office of the Global AIDS Coordinator and Healh Diplomacy (OGAC), and the U.S. Centers for Disease Control and Prevention (CDC). The findings and conclusions in this article are those of the authors and do not necessarily represent the official positions of the funding agencies.
